# Motor-Enriched Learning Activities Can Improve Mathematical Performance in Preadolescent Children

**DOI:** 10.3389/fnhum.2016.00645

**Published:** 2016-12-23

**Authors:** Mikkel M. Beck, Rune R. Lind, Svend S. Geertsen, Christian Ritz, Jesper Lundbye-Jensen, Jacob Wienecke

**Affiliations:** ^1^Department of Nutrition, Exercise and Sports, University of CopenhagenCopenhagen, Denmark; ^2^Department of Neuroscience and Pharmacology, University of CopenhagenCopenhagen, Denmark

**Keywords:** children, motor skills, exercise, integrated physical activity, academic achievement, cognition, learning

## Abstract

**Objective:** An emerging field of research indicates that physical activity can benefit cognitive functions and academic achievements in children. However, less is known about how academic achievements can benefit from specific types of motor activities (e.g., fine and gross) integrated into learning activities. Thus, the aim of this study was to investigate whether fine or gross motor activity integrated into math lessons (i.e., motor-enrichment) could improve children's mathematical performance.

**Methods:** A 6-week within school cluster-randomized intervention study investigated the effects of motor-enriched mathematical teaching in Danish preadolescent children (*n* = 165, age = 7.5 ± 0.02 years). Three groups were included: a control group (CON), which received non-motor enriched conventional mathematical teaching, a fine motor math group (FMM) and a gross motor math group (GMM), which received mathematical teaching enriched with fine and gross motor activity, respectively. The children were tested before (T0), immediately after (T1) and 8 weeks after the intervention (T2). A standardized mathematical test (50 tasks) was used to evaluate mathematical performance. Furthermore, it was investigated whether motor-enriched math was accompanied by different effects in low and normal math performers. Additionally, the study investigated the potential contribution of cognitive functions and motor skills on mathematical performance.

**Results:** All groups improved their mathematical performance from T0 to T1. However, from T0 to T1, the improvement was significantly greater in GMM compared to FMM (1.87 ± 0.71 correct answers) (*p* = 0.02). At T2 no significant differences in mathematical performance were observed. A subgroup analysis revealed that normal math-performers benefitted from GMM compared to both CON 1.78 ± 0.73 correct answers (*p* = 0.04) and FMM 2.14 ± 0.72 correct answers (*p* = 0.008). These effects were not observed in low math-performers. The effects were partly accounted for by visuo-spatial short-term memory and gross motor skills.

**Conclusion:** The study demonstrates that motor enriched learning activities can improve mathematical performance. In normal math performers GMM led to larger improvements than FMM and CON. This was not the case for the low math performers. Future studies should further elucidate the neurophysiological mechanisms underlying the observed behavioral effects.

## Introduction

The acquisition and development of mathematical skills can be seen as a central cognitive attribute in a modern technological society. Successful acquisition of basic mathematical skills early in life provides a framework which later academic achievements are based upon (Duncan et al., 2007), and is a predictor of future academic and professional success (Butterworth, 2005; Parsons and Bynner, 2005). Consequently, it is an important area for researchers in the field of behavioral neuroscience to identify strategies to improve mathematical skill acquisition in children and to explore the mechanisms involved in the acquisition of academic skills.

An emerging line of research has focused on investigating the relationships between physical activity, cognitive functions and academic achievements in children (Hillman et al., 2008; Diamond and Ling, 2016; Donnelly et al., 2016; Pesce and Ben-Soussan, 2016; Vazou et al., 2016; Tomporowski et al., 2015). The term physical activity is a comprehensive concept covering various activities. These activities cover cardiovascular exercise focusing on the quantitative characteristics of the activity (e.g., intensity and duration) aiming at improving the cardiovascular fitness, in addition to activities concerned with the qualitative characteristics of the physical activity (e.g., the coordinative demands and cognitive engagement) leading, for instance, to improved motor skills (Pesce, 2012; Diamond, 2015). Currently, the majority of the performed studies have been concerned with linking cognitive functions (Hillman et al., 2005; Voss et al., 2011) and academic achievements (Castelli et al., 2007; Chaddock-Heyman et al., 2015) to cardiovascular fitness in cross-sectional designs. Recent cross-sectional studies have positively linked motor skills to cognitive and academic measures (Kantomaa et al., 2013; Lopes et al., 2013; Haapala et al., 2014; Geertsen et al., 2016) and recent reviews have stressed the importance of the qualitative characteristics of the performed physical activity as compared to the quantitative characteristics of the physical activity (Best, 2010; Pesce, 2012; Diamond, 2015). In general, however, less focus has been paid to the qualitative characteristics of physical activity, and the relation of these to cognitive functions and academic achievements.

In addition to cross-sectional findings, interventional studies have also focused on the potential of cardiovascular exercise to facilitate cognitive performance and academic achievements (for reviews, see Hillman et al., 2008; Pesce and Ben-Soussan, 2016; Vazou et al., 2016; Donnelly et al., 2016). The theoretical framework for these effects are related to the structural and functional differences and adaptations associated with or resulting from the increased cardiovascular fitness or exercise (Hillman et al., 2008). Indeed, higher-fit preadolescent children display greater gray matter hippocampal volumes (Chaddock et al., 2010), lower gray matter thickness in the frontal cortex (Chaddock-Heyman et al., 2015) and greater white matter integrity (Chaddock-Heyman et al., 2014) which altogether might translate into superior cognitive and academic performance (Chaddock et al., 2010; Chaddock-Heyman et al., 2015). Moreover, interventions focusing on various cardiovascular engaging activities result in brain electrophysiological adaptations, including an increased amplitude of the P3 component of the event-related potentials (ERPs), indicating a more efficient allocation of attentional resources (Polich, 2007). These effects are speculatively based upon neurobiological and molecular events related to cardiovascular exercise which positively affect neuroplastic processes within the central nervous system (Gomez-Pinilla and Hillman, 2013). Again, less focus has been paid to the qualitative characteristics of the physical activity. However, Schmidt et al. (2016) highlighted the promising effects of a cognitively engaging 6-week physical activity intervention in promoting executive functions independently of the exercise intensity. Furthermore, Chang et al. (2013) found a positive effect on measures of cognitive functioning of an 8-week intervention focusing on coordinative demanding intensity-independent physical activity. This positive effect was related to brain electrophysiological measures, including both increased amplitudes and shorter latencies of the P3 ERP component, reflecting more efficient and faster cognitive processing (Polich, 2007).

Altogether, these studies highlight two different approaches of physical activity to facilitate cognitive functions, and suggest differential, but beneficial, mechanisms for the observed behavioral effects (Voelcker-Rehage and Niemann, 2013). This has led to research conducted to investigate the effects of including both quantitatively and qualitatively approaches of physical activity as interventions in ecologically valid school settings to promote cognition and academic achievements. One way of investigating the impact of physical activity on cognition in school settings is to include classroom-based physical activity, prescribed either as physical activity breaks (“energizers”) or physical activity integrated into the academic curriculum. Ahamed et al. (2007) implemented quantitative, cardiovascular “energizers” of 15 min in a 16-month intervention, but did not see any positive effects for the intervention group. Conversely, Mullender-Wijnsma et al. (2016) found that 15-min of classroom-based integrated cardiovascular exercise improved the mathematical and spelling performance to a greater extent than conventional teaching. A paucity of literature exists investigating classroom-based qualitatively focused physical activity. However, a newly published study by Vazou et al. (2016) employed classroom-based integrated multi-faceted physical activity into the taught academic content and found greater improvements in mathematical performance for the intervention group compared to a control group.

Collectively, studies evaluating the effects of classroom-based physical activity (prescribed as breaks or integrated into the curriculum), whether focusing on quantitative or qualitative characteristics, yield inconsistent results. In line with this, Donnelly et al. (2016) recently proposed the need for further research in this specific field to delineate the potential effects. Furthermore, no previous studies have investigated the effects of different qualitatively focused motor enriching interventions (fine vs. gross motor skill) on cognitive and academic performance in children. Recently published cross-sectional study have indeed indicated that different coordinative motor skills, including both gross and fine motor skills, are associated with objective measures of cognitive functions and academic achievement, including mathematical performance, in children (Kantomaa et al., 2013; Haapala et al., 2014; Geertsen et al., 2016). While these associations are intriguing, they are correlational in nature. Therefore, longitudinal interventional studies investigating potential causal effects of integrating different types of qualitative motor activities, including both gross and fine classroom-based motor activities on measures of academic achievements are needed.

Furthermore, a number of studies have aimed at investigating the differential effects of physical activity on cognitive functioning and academic achievement related to the baseline performance of the children (high and low performers). These studies have primarily employed interventions focusing on the quantitative characteristics of the physical activity. Acute cardiovascular exercise interventions have yielded the strongest effects in individuals with the lowest cognitive baseline performance (Mahar et al., 2006; Pontifex et al., 2013; Drollette et al., 2014), highlighting an interesting possibility of using physical activity to support those individuals who need it the most. Yet, the literature regarding the effects of chronic physical activity interventions are sparse. One study that investigated this found that typically developing children benefitted the greatest from a physical education based intervention with additional cognitive demands (cognitive enrichment), while children with coordinative impairments did not (Pesce et al., 2013). This points to the need of optimally challenging every individual. Whether this is the case for classroom-based physical activity interventions is currently unknown. Indeed, studies evaluating classroom-based integrated qualitative physical activity in relation to the children's cognitive and academic baseline performance are lacking.

Additionally, previous studies evaluating the effects of classroom-based physical activity interventions have not accounted for cognitive and motor covariates related to academic achievements, including executive functions (St. Clair-Thompson and Gathercole, 2006; Bull et al., 2008), short-term memory (Raghubar et al., 2010) and motor skills (e.g., Geertsen et al., 2016). Importantly, theoretical models have been proposed, suggesting a mediating role for cognitive efficiency, and particularly executive and metacognitive functions, in the relationship between physical activity and academic achievements (Howie and Pate, 2012; Tomporowski et al., 2015). In line with this, Pesce et al. (2016) recently proposed that improvements in children's ball skills mediated the effects of a coordinative and cognitively demanding physical activity intervention on measures of executive functioning. Moreover, as indicated in a review by Tomporowski et al. (2015) the number of studies investigating the stability and maintenance of the effects resulting from physical activity interventions on measures of cognitive functioning and academic achievement is sparse.

Taken together, the present study has three primary aims: (1) to investigate the immediate and maintained causal effects of classroom-based integrated gross and fine motor activity on mathematical performance in preadolescent children, (2) to investigate whether differences exist in the intervention effects between children characterized as normal and low mathematical performers, and (3) to investigate whether, and to what extent, different cognitive functions and motor skills contribute to the physical activity-academic achievement relationship.

We hypothesize that classroom-based gross and fine motor activity will result in an increased mathematical performance following a 6-week intervention compared to a conventional teaching strategy. We further hypothesize that the addition of extra coordinative demands will primarily benefit normal performing children, in relation to the optimal challenge point theory (Pesce et al., 2013). Finally, based on prior studies highlighting cognitive and motor skills as potential mediators of the effects of physical activity, we hypothesize that both cognitive and motor performance will account for some of the interventional effects on mathematical performance.

## Materials and methods

### Participants

**Figure 1:**
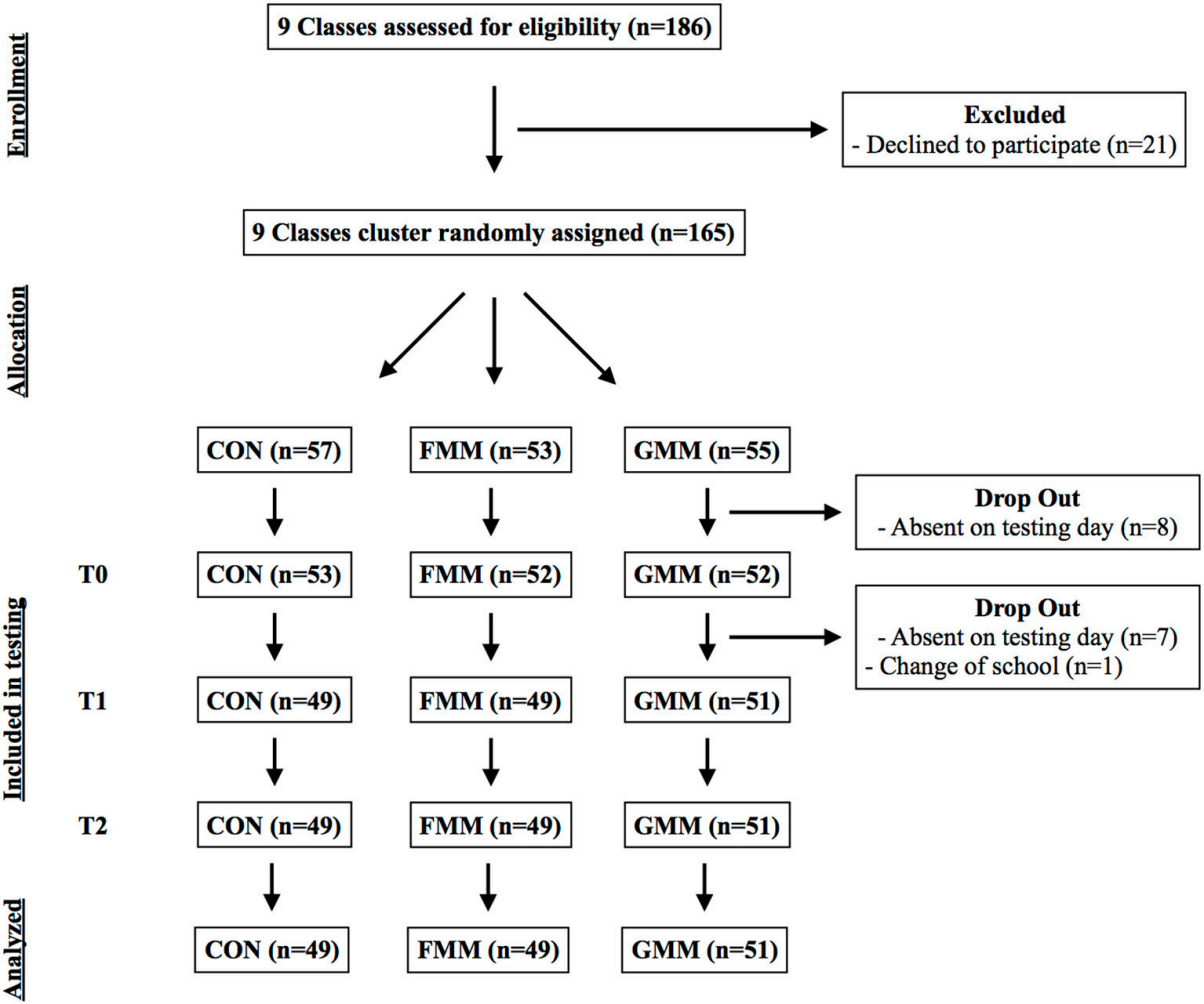
**Flow diagram for the study**.

We invited 186 children from the 1st grade level from three different Danish public schools, containing 9 different school classes, in the Copenhagen area. The schools were selected based on similar demographic profiles (determined by the placement of the schools) and on grade-based graduation performance (included schools ranked 48, 56, 57, of 59 public schools in Copenhagen). One-hundred-sixty-five children (77 girls, mean age = 7.5, SEM = 0.02) were included in the study after obtaining written consent, corresponding to 89% of the invited children (see Table 1 for demographic characteristics within each intervention group). School classes were stratified based on baseline mathematical performance, and cluster-randomly allocated to one of three groups, explained in detail below (Figure 1). The study was approved by the Ethical Committee of Copenhagen, Denmark (protocol: H-15009418), and was carried out in accordance with the Helsinki Declaration II.

**Table 1:** **Demographic characteristics of two intervention groups (FMM, Fine motor math; GMM, Gross motor math) and the control (CON) group**.

	**CON**	**FMM**	**GMM**
Participants (n)	57	53	55
Age (Years)	7.5±0.02	7.5±0.03	7.5±0.02
Gender (% Boys)	49.1±3.8	56.6±3.9	54.5±3.9
Bilingualism (% Bilingual)	36.8±3.7	35.8±3.8	34.5±3.7
BMI (Weight/Height^∧^2)	16.5±0.3	15.8±0.3	16.5±0.3
Cardiovascular fitness (Distance covered, m)	878±17	866±18	894±12

*Data reported as mean ± SEM. Estimate of cardiovascular fitness obtained using the Andersen test (Andersen et al., 2008). No significant between-group differences were observed for any of the measures. BMI, body mass index*.

### Intervention groups

The three groups were gross motor math (GMM), fine motor math (FMM) and control (CON). The groups mainly differed in the applied teaching methods. GMM focused on integrating gross motor movements into the learning activities covering the mathematical curriculum (gross motor enrichment), using different gross motor movements supporting the mathematical principles and procedures to be acquired. Desks and chairs were moved to the sides in the classroom ensuring adequate space for performing gross motor movements. Children in the GMM group performed inter-limb gross motor movements that alternated between dynamic and static movements and involved a large range of movement (e.g., skipping, crawling, hopscotching, throwing, one-legged balance). The gross motor movements were performed while solving mathematical problems throughout all lessons (lasting approximately 60 min each). FMM focused on integrating fine motor movements into the learning activities covering the mathematical curriculum (fine motor enrichment). The learning activities involved a modified version of the LEGO MoreToMath® concept. The children used fine motor activity to manipulate LEGO® bricks supporting the mathematical principles and procedures to be acquired. The children were primarily sitting at their desks throughout the lesson. The children bimanually selected, moved and modeled the bricks using both hands and fingers while solving mathematical problems throughout all lessons (lasting approximately 60 min each). CON employed conventional math teaching, and was restricted to not make use of additional motor activity or cardiovascular exercise during the math lessons. In all three groups (i.e., CON, FMM, GMM), the children worked individually or in small groups during the lessons. During the intervention, the learning activities in the three groups were matched on content, i.e., the mathematical principles and procedures to be acquired, and time of the day of the math lessons. The standardization within and between the intervention groups was ensured through three workshops of 3 h for the involved teachers hosted by the experimental staff. The workshops were conducted prior to the allocation of classes into groups. In addition to thorough instructions, teachers received thoroughly designed teaching manuals, describing what, when and how the teaching should be conducted during the intervention period. Furthermore, the experimental supervisors were in ongoing dialogue with the teachers of all three intervention groups to ensure the intended intervention, however no quantitative measures were obtained during this process.

### Procedure

The children had their mathematical achievement, cognitive functions and motor skills tested at three time-points. Testing took place before (T0), immediately (T1), and 8 weeks (T2) after the intervention. Substantial effort was paid to ensure that the children were tested at the same time of the day ± 1 h, in the same sequence of testing, and by the same experimental supervisor at all three time-points. At T0, the children's cardiovascular fitness was also tested. The intervention lasted 6 continuous weeks, and included 3 weekly math lessons of approximately 60-min duration. During the intervention, the amount and intensity of the physical activity (or load) during the math lessons was evaluated through spot tests involving accelerometers and heart rate monitoring.

### Measures

#### Mathematical test

The children's mathematical achievement was tested through a paper-and-pencil, standardized, diagnostic test developed by experts within the neuropsychological field of mathematical testing in Denmark (Hogrefe Psykologiske Forlag A/S, Virum, Denmark). Specifically, the test consisted of 50 mathematical tasks to be solved, including 39 1st grade tasks and 11 2nd grade tasks. The tasks covered different age-related mathematical themes, including arithmetic and geometry. The test was conducted individually in a classroom with 15–20 children completing it simultaneously under supervision from two experimental supervisors and the teacher of the class. The children were thoroughly instructed prior to the test. During the test, each problem was presented verbally in a standardized format by an experimental supervisor. Next, the children were to solve the presented problems. When the entire classroom had answered, the test proceeded in a similar fashion. Halfway through the test, the children had a 10 min mandatory break. The entire testing session lasted between 60 and 90 min. The tests were reviewed offline by a single experimental supervisor using standardized guidelines provided by the authors of the test.

#### Cognitive tests

Three standardized cognitive tests were applied to estimate the capacity of the children in different domains of cognitive functions, including executive functions and short-term memory. The cognitive tests were completed on a computer in a one-to-one session between a child and an experimental supervisor.

##### Executive functions

The children's executive functions were assessed using a computer-based modified Eriksen Flanker Task (Eriksen and Eriksen, 1974). Since the test was carried out in young children, the stimuli consisted of fish requiring feeding (e.g., Hillman et al., 2014; Schonert-Reichl et al., 2015; Vazou and Smiley-Oyen, 2014). The children were comfortably placed in front of a 15.4″ laptop placed at a distance that allowed them to press the response buttons with their index fingers, with their elbows resting on the edge of the table. The laptop presented the stimuli using Presentation (Neurobehavioral Systems Inc., California, USA). Stimuli (90 × 10 mm) were placed in the center of the screen on a white background. The children were presented with congruent (compatible) stimuli (i.e., > > > > >) and incongruent (incompatible) stimuli (i.e., > > < > >) in a pseudo-randomized sequence with an equi-probable (0.5) frequency of congruent and incongruent stimuli. The children completed a single block of 60 trials, preceded by four familiarization trials, ensuring task compliance. The children were instructed to respond to the inside stimulus, while ignoring the flanking stimuli as fast and accurate as possible. The children's response latency and accuracy were logged for both congruent and incongruent trials. Responses faster than 200 ms were considered as an anticipatory response and were excluded from the analyses. From the congruent and incongruent trials, interference effects (i.e., flanker effects) were computed as the difference between incongruent trials and congruent trials. These measures were used as an estimate of the children's inhibitory control (e.g., Hillman et al., 2009).

##### Visuo-spatial short-term memory

The children's visuo-spatial short-term memory was assessed using a spatial span test from the Cambridge Neuropsychological Test Automated Battery (CANTAB) (Cambridge Cognition Ltd, Cambridge, UK). The spatial span test is a neuropsychological test specifically assessing the memory for sequentially presented visuo-spatial information, and it has been used in previous studies assessing visuo-spatial short-term memory in children aged 4–12 (Luciana and Nelson, 2002). The children were comfortably placed in front of a 23″ touchscreen and equipped with headphones. The touchscreen was placed approximately 30 cm from the edge of the table. During the test, nine white squares (43 × 43 mm each) were presented on a black background on the touch-screen, and the squares changed color one by one. After the presentation, the participants were to replicate the sequence by touching the squares with the index finger of their dominant hand. If correct, an additional color-changing square was added to the sequence in a progressive manner (from 2 to a total of 9 squares). The longest completed sequence, the span length, was logged and used as a measure of the visuo-spatial short-term memory.

##### Phonological short-term memory

The children's phonological (semantic) short-term memory was assessed using a free-recall wordlist memory task inspired by Pesce et al. (2009). Specifically, the test evaluated the children's ability to remember as many words as possible out of 20. The words were all age-appropriate nouns. The children were comfortably placed in front of a 13.3″ laptop and equipped with headphones. During the test, 20 words were visually displayed and presented orally in a standardized and timed sequence for 5-s each using a Microsoft PowerPoint presentation yielding a 100-s presentation time. After the presentation, the children were exposed to a 120-s period in which they were to sit with their eyes closed and remember the words. Then, the children had an additional 120-s period to verbally recall as many words as possible in a free-recall manner. The number of correctly recalled words were logged and used as a measure of the phonological short-term memory.

#### Motor tests

Two motor tasks were applied estimating the children's gross and fine motor skills. The tasks were completed in a one-to-one session between a child and an experimental supervisor.

##### Gross motor skills

To evaluate gross motor skills, the children completed a coordination wall, which has previously been used to assess gross motor coordinative skills in preadolescent children (Geertsen et al., 2016; Larsen et al., 2016). The children were standing facing the coordination wall, which consisted of an upright rectangular 9 × 8 grid, with the numbers 1–10 distributed on the grid. Half of the numbers were blue, half red. Red and blue numbers appeared on both sides of the vertical midline. The coordination wall was split in two by a horizontal dividing line, yielding an upper section (top seven rows), and a lower section (bottom two rows). The children were equipped with a red dot on their right hand and a blue dot on the left hand and foot. The children were instructed to touch the numbers in the correct order, from one to ten, with their hands (upper section) or feet (lower section), according to the color of the number. The movements performed required crossing of the vertical midline. They had to complete the task as fast and accurate as possible. If a mistake was made, the children were immediately instructed to correct it, and proceed. Prior to the test the children were thoroughly instructed. Next, the children had three attempts on the coordination wall, and the shortest time (best time) of completion (in sec) was used as a measure of the children's bimanual, inter-limb coordinative gross motor skills.

##### Fine motor skills

The children's fine motor skills were evaluated using the Perdue Pegboard test (US Neurologicals LLC, Washington, USA). Specifically, the Perdue Pegboard (Tiffin and Asher, 1948) assesses manual dexterity and bimanual fine motor coordination, and has previously been used testing preadolescent children (Gardner and Broman, 1979). The children were comfortably placed on a chair in front of the Purdue Pegboard. Prior to test, the children's handedness were assessed using a name-writing test supplemented with self-report (Scharoun and Bryden, 2014), and the children were thoroughly instructed in the test. The test consisted of four sessions: 30-s uni-manual placement of pins using the dominant hand (i) and the non-dominant hand (ii). Thirteen-seconds bimanual placement of pins using both hands (iii), and 60-s bimanual assembly, consisting of three parts assembled in a specific sequence (iv). The latter served as a measure of the children's fine motor skills, expressed as the number of correctly assembled parts.

#### Cardiovascular fitness

The children's cardiovascular fitness was estimated using the Andersen test (Andersen et al., 2008), which is a validated measure of cardiovascular fitness in 6–9 year old children (Ahler et al., 2012). The test was modified to fit the available space in the gyms of the involved schools. Prior to test, the participants completed a 5-min warm-up and were thoroughly instructed in the test protocol. In the test, the children ran between cones placed diagonally across each other in a distance of 17 m in 15-s intervals interspersed by 15-s mandatory breaks. The test was completed in 10 min. The children ran as fast as they could during the running intervals to cover the greatest distance possible. Heart rate was monitored (Polar Team 2 System, Polar, Finland), and the test was videotaped using a GoPro HERO4 camera (GoPro Inc., California, USA), to ensure that the performance of each child was logged. The videos were inspected offline and the total running distance was registered as a measure of cardiovascular fitness.

#### Physical load during the intervention

In a subsample of the children, covering all three intervention groups (*n* = 49), the physical load during the math lessons was estimated combining time-synced heart rate monitoring (Polar Team 2 System, Polar, Finland) and accelerometers (MinimaxX S4, Catapult Innovations, Canberra, Australia) on 6 occasions. Individual heart rate during the lessons was compared to each participant's maximum heart rate (HRmax) collected during the Andersen test, yielding an individual percent of HRmax. Time spent in low (0–60% of HRmax), moderate-to-vigorous (60–90% of HRmax) and high (90–100% of HRmax) heart rate zones during the lessons were used as outcome measures. The accelerometers sampled tri-axially [forward (fwd), sideways (side), upwards (up)] with a sampling-rate of 100 Hz. Based on the MinimaxX proprietary software (Sprint, Catapult Sports, Canberra, Australia) the physical load (i.e., player load) was computed by the software using the following formula:

$$\begin{array}{l} \text{Player}\;\text{load}=\\ \;\sqrt{{\left(fwd_{y1}-fwd_{y\;-\;1}\right)}^{2}+{\left(side_{x1}-side_{x\;-\;1}\right)}^{2}+{\left(up_{z1}-up_{z\;-\;1}\right)}^{2}} \end{array}$$

where *fwd*_*y*_*, side*_*x*_, *and up*_*z*_ indicate the accelerations in the forward, sideways and upwards plane, respectively. The player load is an arbitrary unit correlated with subjective perceived exertion measures of physical exhaustion (Casamichana et al., 2013), and the measure has previously been used to assess the intensity of various physical activities in preadolescent children (Larsen et al., 2016).

### Statistical analysis

The statistical analyses were performed in the open-access software R Studio (R Core Team, Vienna, Austria). The analyses were carried out on complete datasets (complete-case analysis). Baseline characteristics were compared between groups using one-way analysis of variance or chi-square tests for continuous (age, BMI, cardiovascular fitness) and categorical (gender, bilingualism) measures, respectively. Data from the mathematical, cognitive and motor tests were analyzed using linear mixed models with group-time interactions as fixed effects, using R package *lme4* (Bates et al., 2014). Random effects were included in the models to account for dependencies between measurements on the same subjects, school classes, and schools. Model validation was based upon visual inspection of residual plots and normal probability plots. To accommodate the specific hypotheses of this study, specific sets of contrasts between intervention groups across the included time-points were evaluated using global F-tests. Subsequently, model-based *t*-tests were used to identify the significant differences, using the R-package *multcomp* (Hothorn et al., 2008). These pairwise comparisons were adjusted for multiplicity using the ‘single-step’ adjustment, which is a recently developed procedure providing a less conservative adjustment of *p*-values as compared to Bonferroni adjustment and related adjustments, by utilizing the correlations between tests. Additionally, between-group differences and within-group differences at, and between, specific time-points were compared using model-based *t*-tests. An explorative analysis sought to examine whether differences between intervention groups were related to the baseline mathematical performance level of the children. The authors of the standardized and validated math test describe that an individual performance at 75% correct answers, or lower, might reflect difficulties acquiring mathematical content. This subgroup was termed *low performers* (*n* = 49, based on the math performance at T0). The other subgroup (i.e., >75% c.a.) was termed *normal performers* (*n* = 116) and was characterized by not having difficulties acquiring mathematical content. Moreover, to gauge the tentative contribution or mediating effect of cognitive and motor performance on mathematical achievement, each cognitive and motor measure was added one by one, using an univariate approach, to the overall linear mixed model as an additional covariate, and differences in estimates between models with and without the covariate were reported. Data are reported as mean ± SEM unless otherwise stated. A significance level of 0.05 was applied.

## Results

### Baseline mathematical, cognitive, and motor performance

At T0, the groups performed equally well in the measures of mathematical and visuo-spatial short-term memory performance, in addition to gross and fine motor performance. This is presented in Table 2. Indeed, model-based *t*-tests revealed no significant between-group differences in these measures (*p* > 0.05). However, significant between-group differences at T0 were found in measures of inhibitory control and phonological short-term memory (Table 2). FMM performed significantly better in the accuracy interference score compared to GMM (*p* = 0.01), and CON performed significantly worse than FMM (*p* = 0.049) and GMM (*p* = 0.03) at T0 in phonological short-term memory.

**Table 2:** **Mathematical, cognitive and motor performance at T0, T1 and T2 for the two intervention groups (FMM, Fine motor math; GMM, Gross motor math) and the control (CON) group**.

**Measure**	**CON**			**FMM**			**GMM**		
	**T0**	**T1**	**T2**	**T0**	**T1**	**T2**	**T0**	**T1**	**T2**
**MATHEMATICAL PERFORMANCE (*N* = 149)**									
Math score (No. correct answers)	36.1±1.3	38.9±1.3^*^	39.2±1.2^*^	37.3±1.2	39.1±1.2^*^	40.4±1.1^*^§	36.8±1.2	40.6±1.2^*^	41.1±1.1^*^
**VISUO-SPATIAL SHORT-TERM MEMORY (*N* = 139)**									
Span length (No. boxes)	5.0±0.2	5.1±0.2	5.6±0.2^*^§	4.8±0.2	5.2±0.2	5.9±0.2^*^§	4.8±0.2	5.5±0.2^*^	5.8±0.2^*^
**PHONOLOGICAL SHORT-TERM MEMORY (*N* = 136)**									
Words recalled (No. words)	5.4±0.5^b^^,^^c^	5.7±0.5	5.4±0.5	6.4±0.5	5.7±0.5	6.6±0.4^§^	6.3±0.4	5.1±0.4^*^	6.3±0.4^§^
**EXECUTIVE FUNCTIONS (*N* = 134)**									
Accuracy (% correct): congruent trials	94.6±0.8	96.6±0.8	97.2±0.7^*^	97.1±0.8^a^	98.9±0.8	98.1±0.7	96.5±0.8	97.7±0.8	97.7±0.7
Accuracy (% correct): incongruent trials	93.4±1.1	95.0±1.1	95.0±1.0	96.3±1.0^c^	97.6±1.0	96.9±1.0	92.6±1.0	95.7±1.0^*^	94.4±1.0
Response latency (ms): congruent trials	802±44	733±44^*^	707±33^*^	861±43	704±43^*^	657±33^*^	851±42	723±42^*^	692±32^*^
Response latency (ms): incongruent trials	863±61	763±61^*^	743±48^*^	927±61	753±61^*^	700±48^*^	943±60	800±60^*^	756±47^*^
Interference effect: accuracy (% correct)	−1.3±0.9	−1.6±0.9	−2.2±0.8	−0.7±0.9^c^	−1.3±0.9	−1.2±0.9	−3.9±0.9	−2.0±0.9	−3.2±0.8
Interference effect: response latency (ms)	62±23	31±23	38±21	66±23	49±23	43±21	92±23	78±23	66±21
**GROSS MOTOR PERFORMANCE (*N* = 136)**									
Best time (s)	20.9±0.7	17.6±0.7^*^	15.7±0.9^*^§	20.0±0.7	17.9±0.7^*^	14.7±0.8^*^§	21.5±0.7	17.7±0.7^*^	15.2±0.8^*^§
**FINE MOTOR PERFORMANCE (*N* = 126)**									
Assembly (No. of parts)	20.1±1.7	21.5±1.7^*^	24.9±1.6^*^§	21.6±2.0	22.7±2.0	27.9±1.9^*^§	21.3±1.6	22.8±1.6^*^	25.6±1.6^*^§

*Data reported as means ± SEM. Mathematical performance estimated by standardized, diagnostic Danish test. Visuo-spatial and phonological memory estimated using a spatial span and a word-recall task. Executive functions estimated using a modified Eriksen Flanker Task. Interference effect is the difference between incongruent and congruent trials. Gross and fine motor performance evaluated through a coordination wall task and the Perdue Pegboard, respectively*.

* *Indicates a significant within-group difference from T0*.

§ *Indicates a significant within-group difference from T1*.

a *Indicates a significant between-group difference from CON at T0*.

b *Indicates a significant between-group difference from FMM at T0*.

c *Indicates a significant between-group difference from GMM at T0 (p < 0.05)*.

### Physical load and attendance during the intervention

To evaluate the physical load of the math lessons during the intervention we applied combined accelerometer and heart rate measures at 6 randomly selected math lessons. Furthermore, we recorded the attendance in the math lessons during the intervention. Group means of the physical load during the math lessons as well as the attendance during the intervention are presented in Table 3. No between-group difference was found in the measure of attendance [*F*_(2, 161)_ = 0.51; *p* = 0.60], indicating homogeneity in the attendance between groups during the intervention. An overall significant between-group difference was found for player load [*F*_(2, 48)_ = 27.2, *p* < 0.001], and revealed that GMM displayed a higher player load during the math lessons compared to both FMM (*t* = 6.33, *p* < 0.001) and CON (*t* = 6.13, *p* < 0.001). A significant group-zone interaction was found for time spent in different heart rate zones [*F*_(2, 138)_ = 4.58, *p* = 0.002], and revealed that GMM spent more time in the moderate-to-vigorous heart rate zone (60–90% of HRmax) compared to both FMM 10.0 ± 4.0% (*p* = 0.03) and CON 10.6 ± 4.1 % (*p* = 0.03). These results indicate that GMM performed significantly more accelerations during the math lessons in the intervention, and spent more time in the moderate-to-vigorous heart rate zone compared to both FMM and CON.

**Table 3:** **Attendance and physical load during the intervention for the two intervention groups (FMM, Fine motor math; GMM, Gross motor math) and the control (CON) group**.

	**CON**	**FMM**	**GMM**
**ATTENDANCE (*N* = 165)**			
Attended math lessons (%)	90.9±1.4	92.7±1.2	91.3±1.3
**PHYSICAL LOAD (*N* = 49)**			
Time spend in low HR zone (%)	88.8±3.1^c^	87.9±2.9^c^	77.9±2.7^a^^,^^b^
Time spend in MVPA HR zone (%)	11.1±3.1^c^	11.7±2.9^c^	21.6±2.7^a^^,^^b^
Time spend in high HR zone (%)	0.2±3.1	0.4±2.9	0.4±2.7
Player Load/min (a.u.)	0.07±0.006^c^	0.07±0.006^c^	0.12±0.007^a^^,^^b^

*Data reported as means ± SEM. Physical load obtained through six spot tests using time-synced heart rate measures and accelerometers. MVPA, moderate-to-vigorous physical activity; HR, Heart rate. Low HR zone corresponds to 0–60% of HR_max_, MVPA HR zone corresponds to 60–90% of HR_max_ and high HR zone corresponds to 90–100% of HRmax*.

a *Indicates a significant between-group difference from CON at T0*.

b *Indicates a significant between-group difference from FMM at T0*.

c *Indicates a significant between-group difference from GMM (p < 0.05)*.

### Performance in the mathematical test

**Figure 2:**
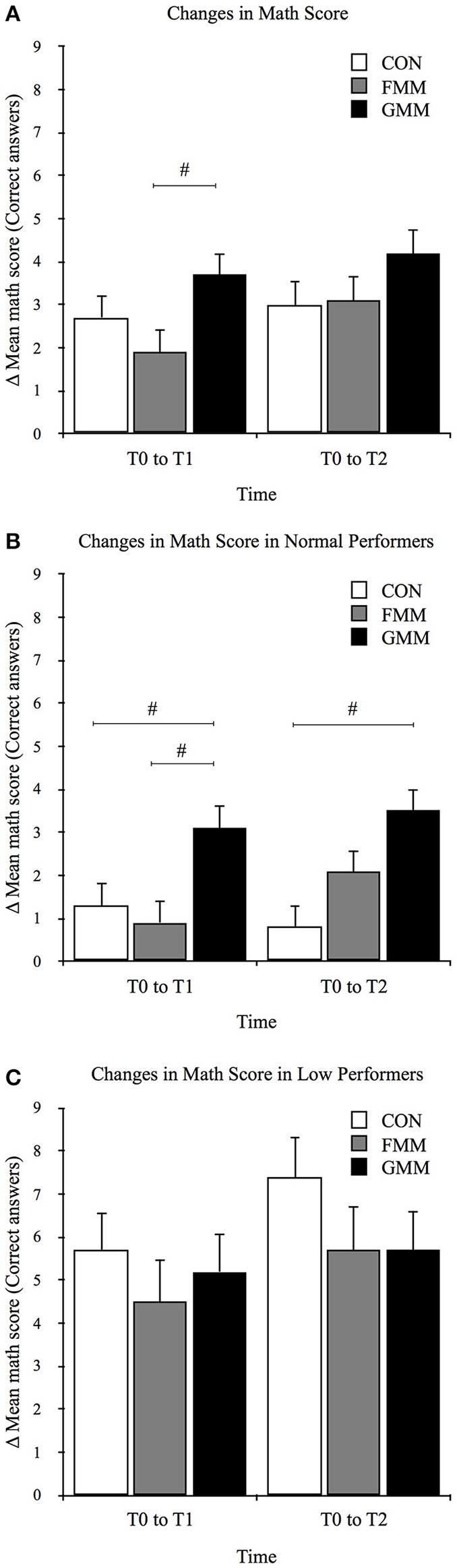
**Changes in mathematical performance**. Displays changes in mathematical performance expressed as means ± SEM from T0-T1 and T0-T2 in **(A)** all children, **(B)** normal performers (≥75% c.a. in 1st grade tasks at T0), **(C)** low performers (≥75% c.a. in 1st grade tasks at T0). CON, Control; FMM, Fine motor math; GMM, Gross motor math. # indicates a significant between-group difference in the improvements in mathematical performance between time points (*p* < 0.05).

All groups improved their performance within the mathematical task from T0 to T1 and T0 to T2, as can be seen in Table 2. A significant group-time interaction was found from T0 to T1 [*F*_(2, 288)_ = 3.49, *p* = 0.03]. As summarized in Figure 2A, the changes in mean mathematical performance were significantly greater from T0 to T1 for GMM compared to FMM 1.87 ± 0.71 correct answers (c.a.) (*p* = 0.02). This effect was not evident from T0 to T2 [*F*_(2, 434)_ = 1.89, *p* = 0.15]. This indicated that GMM transiently improved children's performance in the mathematical task more than FMM.

### Performance in the mathematical test in normal and low performers

To assess whether differences existed between children characterized as normal and low mathematical performers we performed a subgroup analysis. Within the normal performers, the improvements in mean mathematical performance were significantly greater for GMM compared to both CON 1.78 ± 0.73 c.a. (*p* = 0.04) and FMM 2.14 ± 0.72 c.a. (*p* = 0.008) from T0 to T1 (Figure 2B). Additionally, the improvements in mean mathematical performance were significantly greater for GMM compared to CON 2.67 ± 0.71 c.a. (*p* < 0.001) from T0 to T2 (Figure 2B). No significant differences between groups were observed within the low performing individuals from T0 to T1 or T0 to T2 (all *p* > 0.05) (Figure 2C). This indicates that the normal performers benefitted from GMM compared to both FMM and CON, whereas the low performers did not.

### Contribution of cognitive and motor covariates to changes in mathematical performance

Additionally, we investigated whether, and to what extent, potential covariates contributed to the results observed for the mathematical performance, by using an univariate analysis. Group means of the cognitive and motor performance measures can be seen in Table 2. The results of the univariate analysis are presented in Table 4. When controlling for visuo-spatial short-term memory the difference in mathematical performance between GMM and FMM was reduced to 1.22 ± 0.8 (*p* = 0.40). Changes in visuo-spatial short-term memory accounted for approximately 35% of the effects of the intervention on mathematical performance. Similar results were not found for any other measure of cognitive performance (see Table 4). When controlling for gross motor skills the difference in mathematical performance between GMM and FMM was reduced to 1.41 ± 0.77 (*p* = 0.16). Changes in gross motor skill performance accounted for approximately 25% of the effects of the intervention on mathematical performance (see Table 4).

**Table 4:** **Estimated between-group differences (GMM vs. FMM) with cognitive and motor covariates**.

	**Estimated between-group difference**	**Percent contribution**
Without covariate	1.87 ± 0.7	
Phonological short-term memory (No. words)	1.81 ± 0.7	3.2
Visuo-spatial short-term memory (No. boxes)	1.22 ± 0.8	34.8
Executive functions (Interference effect, accuracy %)	1.94 ± 1.0	−3.7
Executive functions (Interference effect, RT in ms)	1.99 ± 1.0	−6.4
Fine motor skills (No. of parts)	1.67 ± 0.9	10.7
Gross motor skills (Best time, in sec)	1.41 ± 0.8	24.6

*Data reported as means ± SEM. Different covariates added to the statistical model explaining intervention effects in mathematical performance. Percent contribution displays percent accounted for by a single covariate. The intervention effects are primarily accounted for by visuo-spatial short-term memory and gross motor skills*.

## Discussion

The aims of this study were 3-fold. First, we sought to investigate the immediate and longer-term effects of classroom-based integrated gross and fine motor activities on mathematical achievement in preadolescent children. Secondly, it was investigated whether the intervention elicited different effects in normal and low math performers. Thirdly, we sought to investigate the potential role of cognitive functions and motor skills in the physical activity-academic achievement relationship.

The main findings of the study were that motor enriched learning activities can improve mathematical achievement. Indeed, in normal performers, applying gross motor enriched math lessons resulted in a greater improvement in mathematical performance compared to fine motor enriched math and conventional math lessons after a 6-week intervention. The effects on mathematical performance were maintained between gross motor enriched and conventional teaching in the normal performers 8 weeks after the cessation of the intervention. In all children, these positive effects were observed between gross motor enriched learning and fine motor enriched learning. These effects seem to be partly accounted for by changes in the visuo-spatial short-term memory, gross motor skills and, to a minor degree, fine motor skills. These results add to the emerging literature, consolidating the positive effects of integrating classroom-based motor activity to improve cognitive and academic performance in children (Donnelly et al., 2016). Interestingly, our results specify that only normal achieving children benefit from adding motor activity to the classroom curriculum, and that cognitive and motor abilities could contribute to the observed positive effects of the motor enriched learning activities.

### Effects of gross motor-enriched learning activities on mathematical performance

The observed behavioral effects might be the result of a combination of different mechanisms. Indeed, a recently published review proposed that classroom-based integrated physical activity could influence learning through various processes (Chandler and Tricot, 2015), including acute and chronic effects of the physiological response to exercise on the central nervous system (CNS) (Hillman et al., 2008). For example, physical exercise causes a release of a plethora of neurobiological substances, including brain-derived neurotrophic factor (BDNF), lactate and insulin-like-growth factor (IGF-1) (Skriver et al., 2014). Animal studies suggest that these neurotrophic agents might benefit neuroplastic processes, acutely related to memory formation (e.g., Cotman and Berchtold, 2002; Cotman et al., 2007). However, previous studies have demonstrated an intensity-dependent dose-response relationship of exercise on the neurotrophic response (Ferris et al., 2007; Winter et al., 2007), leading to the greatest physiological response at intensities higher than the one experienced in the gross motor enriched learning activities in the current study (22% in moderate-to-vigorous heart rate zone). Additionally, a direct transfer of the animal-based molecular findings to human behavioral outcomes is irrefutably challenging (Voss et al., 2013). Indeed, while some human studies have found associations between blood concentrations of a number of neurotrophic agents and behavioral outcomes (Winter et al., 2007; Skriver et al., 2014), others have failed (Ferris et al., 2007; Schmidt-Kassow et al., 2013).

At a functional and behavioral level in children, single bouts of moderate-to-vigorous physical activity have been related to efficient and rapid allocation of attentional resources (Hillman et al., 2009) and improved classroom behavior (Mullender-Wijnsma et al., 2015). Results from chronic intervention studies focusing on moderate-to-vigorous physical activity have also pinpointed these positive effects (e.g., Hillman et al., 2014). Moreover, single bouts of coordinatively demanding physical activity have been found to improve attention in adolescents (Budde et al., 2008), and chronic interventions employing coordinatively demanding physical activity have successfully improved children's attention (Chang et al., 2013; Gallotta et al., 2015). Attention is a key mediator of hippocampal-related declarative memory formation (Aly and Turk-Browne, 2016), probably related to schema-dependent academic learning and performance (van Kesteren et al., 2014).

We hypothesized a general effect of motor enriched learning strategies. Yet, the gross motor learning activities were the single effective strategy in promoting mathematical performance. Arguably, the greater time spent in moderate-to-vigorous physical activity during the gross motor enriched learning activities, working in conjunction with greater coordinative demands, could have favored brain processes positively contributing to the effects of gross motor enriched learning activities on both an acute (during the lessons) and chronic (throughout the entire intervention) temporal scale.

Additionally, based on theories of embodied cognition (e.g., that cognitive knowledge is based on bodily experiences) (Barsalou, 2008), learning could be influenced by integrating task-related motor activity, bridging the content to be acquired to the performed motor activity. Indeed, procedural sensorimotor experiences might contribute to declarative knowledge acquisition (Koziol et al., 2012). Moreover, neuroanatomical structures, including the cerebellum, previously thought to be primarily motor related might also be critically involved in controlling higher-order cognitive functions (Diamond, 2000; Koziol et al., 2012). Previous studies have indeed found a positive effect of performing movements related to the content to be acquired in academically related domains in both adults (Macedonia et al., 2011; Mayer et al., 2015) and young children (Mavilidi et al., 2015). Collectively, these studies demonstrate the positive effects of performing congruent motor activity to improve learning. Theoretically, it could be speculated that the gross motor enriched learning activities involved motor activity more likely to be subjectively perceived as more figuratively meaningful and congruent, compared to the motor activity performed during the fine motor enriched learning activities. This was, however, not assessed during the study.

Taken together, while both the quantitative (e.g., exercise intensity), qualitative (e.g., coordinative demands) and embodied (e.g., congruency) characteristics of the physical activity might explain the behavioral differences observed, more research is needed to pinpoint the exact mechanisms underlying the effects.

### Differential effects of interventions on mathematical performance related to the baseline level of the children

The results obtained in our subgroup analyses showed that the benefits of gross motor enriched learning activities were confined to the normal performing individuals. The low-performing children generally improved their mathematical performance more. However, no differences were observed in the improvements between groups. This was in contrast to the normal performing individuals, where benefits of gross motor enriched learning activities were present. Previous research have, on the other hand, shown that the effects of physical activity on cognition and academic involvement were greatest for low-performing children (Mahar et al., 2006; Drollette et al., 2014). However, whereas this study included measures of mathematical performance in a longitudinal interventional perspective, previous studies have used measures of executive functioning (Drollette et al., 2014) and class-room behavior (Mahar et al., 2006) in acute study designs. Furthermore, these studies all employed physical activity interventions centered around the quantitative characteristics of the performed activity. Moreover, the results of the abovementioned studies might be partially biased by the statistical phenomenon of regression toward the mean, explaining the tendency for an “extreme” measure to be closer to the mean when measured the second time (e.g., Moreau et al., 2016). In contrast, the results of the current study do not seem to be biased by this statistically observable phenomenon, as it was within the normal performing subgroup that the benefits of gross motor learning activities were observed. Taken together, the parameters mentioned above complicate direct comparisons to the results of the current study.

Importantly, however, our results contribute with novel knowledge clarifying who benefits from classroom-based integrated motor activity. It seems that the combined cognitive and motor demands of the gross motor enriched teaching strategies result in positive effects uniquely for normal performers, and not for the low performers. These findings support the notion of an optimal challenge point, as initially proposed by Pesce and colleagues (Pesce et al., 2013). Specifically, Pesce et al. (2013) found that enriching a physical activity intervention with additional cognitive demands specifically centered around the executive functions of the participants, resulted in greater improvements in measures of flexible attention for typically developing children, but not for children with developmental coordinative motor deficits. The authors argued that children with motor deficits required a higher amount of cognitive control merely performing the physical activity, leaving less cognitive resources available to deal with additional cognitive challenges (Pesce et al., 2013). In line with this, one could speculate that the low-performing individuals were sufficiently challenged by the cognitive demands of the mathematical content to be acquired during the lessons, due to their initial lower mathematical skill proficiency, leaving fewer mental resources available to benefit from the additional motor activity posed by the gross motor enrichment. Collectively, enriching mathematical lessons with gross motor activity seems to be optimal for normal performing individuals, but not for low performing individuals.

Despite these interesting findings, the current evidence in the field does not allow for clear conclusions regarding the responsiveness of physical activity interventions in individuals achieving at different levels at baseline. Future research is needed to investigate the potential inter-individual differences in the effectiveness of interventions aiming at improving academic achievements in children, using studies specifically designed for evaluating this question.

### Visuo-spatial short-term memory and gross motor skill performance accounts for mathematical improvements

The performed univariate covariation analysis arguably provides novel, interesting perspectives on the effects of physical activity on academic achievement, by showing that changes in visuo-spatial short-term memory in addition to gross motor skill performance partially accounted for the effects of the intervention on mathematical performance. This was not the case, to the same extent, for the other included cognitive or motor measures, indicating specific associations between physical activity, visuo-spatial memory, gross motor skills and mathematical achievement. In support of this, visuo-spatial memory has previously been related to mathematical achievements (Bull et al., 2008), especially in young children (Holmes and Adams, 2006). Moreover, previous cross-sectional findings have supported a relationship between measures of fine and gross motor proficiency and cognitive functions in both elderly (Voelcker-Rehage et al., 2010) and children (Geertsen et al., 2016). A non-interventional longitudinal study also found that motor skills in kindergarten predicted academic achievement in 1st grade children (Roebers et al., 2014). The results of the current study add longitudinal and interventional evidence to the current knowledge, suggesting that gross motor enriched learning activities might improve mathematical performance through improved gross motor skills and visuo-spatial short-term memory. These results fit nicely with the only other developmental study addressing the potential mediating role of gross motor skills, but not fine motor skills, on executive functioning (Pesce et al., 2016), which is closely related to mathematical performance in children (St. Clair-Thompson and Gathercole, 2006). However, the causal interactions between the performance measures included in the current study are difficult to infer from these results, and the results should be seen as an initial exploration of possible mediating effects. Previous studies applying classroom-based interventions to improve academic achievements through physical activity have not controlled for the contribution of covariates to the same extent as in the current study. Thus, the novelty of the current findings warrants the need for investigating the contribution of cognitive and motor covariates when evaluating the effects of physical activity on academic achievements in future studies.

### Strengths and limitations

This study was strengthened by the ecological value of the design including school classes as the level of randomization in the cluster-randomized controlled trial. In addition, by controlling what, when and how the participants were taught while still keeping their regular teachers and framework, we ensured that these factors did not influence our results substantially. Moreover, we evaluated the long-term effects caused by the intervention, which adds extremely important knowledge about the stability of the intervention. However, some limitations should also be kept in mind when interpreting the results of the study. First, the combination of a relatively small sample size and a substantial intra- and inter-individual variability might have influenced the power of the study. A larger sample size could strengthen the effects of the intervention (Gelman and Carlin, 2014; Moreau et al., 2016). Additionally, we acknowledge that the test measures included in the study are subject to practice-effects (i.e., test-retest effects). However, these would expectedly affect the included groups equally, and hence would not bias the inter-group comparisons on which our conclusions are based. Even though we included various objective measures of motor skills and cognitive functions as potential covariates affecting the effects of the intervention on mathematical performance, our study could also have included measures of motivation, social interactions during the lessons and mental well-being as recently pointed out in the review by Diamond and Ling (2016) to strengthen the interpretation of the results. Indeed, integrating physical activity into the classroom have been found to influence motivational aspects (e.g., Vazou and Smiley-Oyen, 2014; Vazou and Skrade, 2016). Moreover, the results of our univariate approach did not account for collinearity between predictors and, with this shortcoming in mind, they should be seen as an initial exploration of possible mediating effects. However, more sophisticated techniques for mediation analysis, beyond the scope of the present study, such as the one applied by Pesce et al. (2016), would allow a more comprehensive exploration while accommodating for collinearity. Inferring the exact mechanisms underlying the observed behavioral effects in the current study design is challenging. Future studies should investigate these. Structural and functional imaging techniques could prove a valuable tool to widen our understanding of the underlying mechanisms.

## Conclusion

Participation in math lessons focusing on integrating gross motor activity can positively contribute to mathematical achievements in preadolescent children. In normal math performers, gross motor enrichment led to larger improvements than fine motor enrichment and conventional teaching. Across all children gross motor enrichment resulted in greater mathematical achievement compared to fine motor enrichment. From a practical perspective, teachers and related personnel should consider integrating gross motor activity in learning activities relevant to the academic curriculum as a promising way to engage children and improve academic achievement. The subgroup differences suggest the need of individually tailored teaching activities, specifically related to the individuals' optimal challenge point, to enhance learning in academic domains in children.

## Author contributions

MB, RL, JW, SG, and JL designed the experiment. MB, RL, and JW collected the data. MB, RL, JW, SG, and CR conducted the required data analysis. All authors contributed to drafting the manuscript, and all authors approved the final version of the manuscript.

## Funding

This project was supported by a grant from the LEGO-foundation. LEGO-Education provided the MoreToMath® products.

### Conflict of interest statement

The authors declare that the research was conducted in the absence of any commercial or financial relationships that could be construed as a potential conflict of interest. LEGO-Education provided the MoreToMath® products, otherwise they were not involved before, during or after the project.
